# Directing HIV-1 for degradation by non-target cells, using bi-specific single-chain llama antibodies

**DOI:** 10.1038/s41598-022-15993-y

**Published:** 2022-08-04

**Authors:** Jord C. Stam, Steven de Maat, Dorien de Jong, Mathia Arens, Fenna van Lint, Lavina Gharu, Mark H. van Roosmalen, Rob C. Roovers, Nika M. Strokappe, Ralf Wagner, Alexander Kliche, Hans J. de Haard, Paul M. van Bergen en Henegouwen, Monique Nijhuis, C. Theo Verrips

**Affiliations:** 1 Cell Biology, Neurobiology and Biophysics, Department of Biology, Science Faculty, Utrecht University, 3584 CH Utrecht, The Netherlands; 2 Translational Virology, Department of Medical Microbiology, University Medical Center Utrecht, Utrecht, The Netherlands; 3 Molecular Microbiology and Gene Therapy, Institute of Medical Microbiology and Hygiene, University of Regensburg, Regensburg, Germany; 4 Argenx, Industriepark Zwijnaarde 7, 9052 Zwijnaarde, Belgium; 5 QVQ Holding BV, Yalelaan 1, 3584 CL Utrecht, The Netherlands; 6 Present Address: Intervet, Wim de Körverstraat 35, 5831 AN Boxmeer, The Netherlands; 7 Present Address: LAVA Therapeutics, Yalelaan 60, 3584CM Utrecht, The Netherlands

**Keywords:** Mechanisms of disease, Infectious diseases, Molecular medicine

## Abstract

While vaccination against HIV-1 has been so far unsuccessful, recently broadly neutralizing antibodies (bNAbs) against HIV-1 envelope glycoprotein were shown to induce long-term suppression in the absence of antiretroviral therapy in patients with antibody-sensitive viral reservoirs. The requirement of neutralizing antibodies indicates that the antibody mediated removal (clearance) of HIV-1 in itself is not efficient enough in these immune compromised patients. Here we present a novel, alternative approach that is independent of a functional immune system to clear HIV-1, by capturing the virus and redirecting it to non-target cells where it is internalized and degraded. We use bispecific antibodies with domains derived from small single chain Llama antibodies (VHHs). These bind with one domain to HIV-1 envelope proteins and with the other domain direct the virus to cells expressing epidermal growth factor receptor (EGFR), a receptor that is ubiquitously expressed in the body. We show that HIV envelope proteins, virus-like particles and HIV-1 viruses (representing HIV-1 subtypes A, B and C) are efficiently recruited to EGFR, internalized and degraded in the lysosomal pathway at low nM concentrations of bispecific VHHs. This directed degradation in non-target cells may provide a clearance platform for the removal of viruses and other unwanted agents from the circulation, including toxins, and may thus provide a novel method for curing.

## Introduction

Human Immunodeficiency Virus (HIV) is the cause of acquired immunodeficiency syndrome (AIDS). In spite of considerable efforts, until now no successful vaccine against HIV-1 has been developed^1,2^. Fortunately, the discovery of combination antiretroviral therapy (cART) resulted in effective suppression of viral replication and substantially reduced AIDS-related morbidity and mortality. However, cART does not eliminate HIV that persists in a latent state, and it also cannot stop viral production from these latent reservoirs. Treatment interruption results in renewed viral replication and a rapid viral rebound from these latent reservoirs^3–5^. For this reason, lifelong daily adherence to cART is required in infected individuals, which is costly, associated with side effects and can result in selection of multidrug-resistant escape mutants.

HIV-1 specific broadly neutralizing antibodies (bNAbs) present an immune therapy based alternative for cART^6–12^. bNAbs have been isolated from the sera of some HIV-1 infected individuals. These individuals have developed the bNAbs in a later phase of infection and the bNAbs bind to one of several conserved epitopes present on the envelope glycoprotein gp120, thereby inhibiting viral entry into the target cells. Administration of bNAbs to other patients was shown to promote suppression i.e. control of plasma viremia in the absence of cART. Interestingly, they may also facilitate virus clearance from the infected cells^7,10^.

The conserved epitopes to which the bNAbs bind, comprise parts of the envelope proteins that have essential roles in the infection process. For example, the VRC01- class is the subset of bNAbs that binds to the conserved CD4 receptor binding sites (CD4bs) on gp120, which is reported to neutralize all major circulating subtypes of HIV-1 by partially mimicking the binding of CD4^13^. Studies in non-human primates with VRC01 administration report protection against SIV/HIV challenges^14^, and also in humans VRC01 administration results in reduction in acute viremia^15^ and limited viral reservoir establishment^16,17^. Moreover, in HIV-1 infected individuals undergoing analytical treatment interruption (ATI) of cART, bNAbs can maintain suppression for weeks, the length of suppression being related to their relative neutralization potency^10^. However, the major problem using single bNAb is the emergence of antibody resistant viral variants**,** as has been reported by several bNAb clinical trials conducted so far^8,13,15,18,19^. Focus has then shifted to using a combination of bNAbs, very similar to the use of combination ART^9,11,20^. The combination of anti-HIV-1 monoclonal antibodies 3BNC117 and 10–1074 can maintain long term suppression in the absence of cART. However, they are only applicable in individuals with antibody sensitive viral reservoirs^10^ and efficacy may get reduced by a high mutation rate of HIV. Also, upon binding of the antibodies the rate of actual clearing of HIV viruses is not well defined and is most likely compromised in HIV patients, due to evolutionary adaptation of the virus.

In this study we present an alternative way to clear HIV viral particles, making use of bispecific antibodies (BsAbs) that have different functional domains combined in one antibody molecule. Since the first reports about recombinant BsAbs using diabodies^21^ or IgG linked to a scFv^22^, the rational design of BsAbs has greatly improved and has found applications in autoimmune diseases, cancer and infectious diseases^23–26^, as well as in improving HIV treatment^27–29^^.^. Camelids make beside conventional IgG a special type of antibodies, heavy chain only IgG, of which the antigen binding properties reside in a small single chain variable domain^30–32^. This domain can easily be cloned and expressed in microorganisms, resulting in 15 kD antibodies called VHH antibody fragments, also referred to as Nanobodies (trademarked by Ablynx). Immunizations of llamas with antigens generally result in very good immune responses in both the conventional class of IgG and in the heavy chain-only class. By phage display methods HIV- binding and neutralizing VHHs can efficiently be retrieved^33–37^. The VHH binding domains are particularly suitable for construction of bivalent, bispecific binding molecules, since multiple VHHs can easily be combined in one molecule^26,31,32,38^. The VHH technology has a number of favorable properties compared to conventional and other recombinant antibody technologies including small size, improved tissue penetration, high solubility, good stability, ease of genetic manipulation and relative cheap production cost when produced in microorganisms^31,32,39^. Using these favorable properties, we have developed a novel type of bispecific VHHs that can be used for viral clearance independent of a functional immune system. These bispecific VHHs consist of a domain recognizing HIV-1 and the other domain recognizing EGFR, a cell surface protein that is ubiquitously expressed on many cell types of the human body. The bispecific antibodies are five times smaller than conventional antibodies. Thus, with the same amount of protein a large number of combinations of different antibodies can be applied to minimize the risk of escape mutants. We show here in tissue culture that the bispecific VHHs can bind HIV-1 envelope glycoprotein and deliver it to EGFR in cells upon which the virus antigen–antibody complex is internalized and degraded by the lysosomal degradation pathway. In vivo this may actively clear the virus from the body.

## Results

### Construction and functionality of bi-specific bi-head VHHs

Previously we and others have generated anti-HIV-1 VHHs (small llama antibody fragments), yielding a considerable number of VHHs with broad binding capacity, including binders with blocking/neutralization activity^33–37,40^. In the present study we investigated a novel approach by construction of bispecific VHH antibodies that are able to direct degradation of HIV-1 by non-target cells. We employed previously generated VHHs binding to the ectodomain of EGFR, including VHHs that were internalized after binding to EGFR^41–44^. We combined four different anti-HIV-1 VHHs (1F10, H3, 2H10, 2E7)^35,37,40^ indicated here as H2, H3, H4 and H5 with two different anti-EGFR VHHs (EgA1, EgB4) indicated as E1 and E2. The anti-HIV-1 VHHs are specific for different envelope glycoprotein epitopes and were chosen on basis of broad binding capacity for different clades of HIV-1, including clades A, B and C. The E1, E2 VHHs bind two different epitopes on the EGFR ectodomain with high affinity^41,42^. Table 1 shows the bispecific VHH domains used and summarizes their characteristics. The bispecific VHHs (biheads) were generated by PCR—cloning and contain a flexible linker sequence of 10 amino acids [(G_4_S)_2_].

**Table 1:**
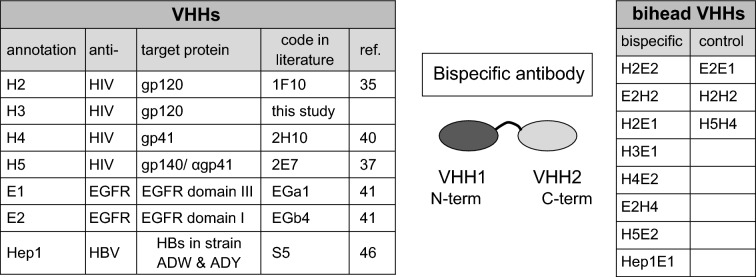
VHH llama antibodies combined in bispecific VHHs with a sequence of [G_4_S]_2_ in between the VHHs.

H is anti-HIV-1, E is anti-EGFR and Hep is anti-hepatitis B VHH. Bispecific VHHs were generated in various combinations. E.g. H2E2 denotes a bispecific VHH antibody with N-terminal VHH 1F10 linked to C-terminal EGb4. E1 (EgA1) is an antagonistic anti-EGFR VHH that blocks EGF binding, whereas E2 (EgB4) is a non-antagonistic antibody that binds to Domain I of EGFR^41^. The anti-HIV antibodies H2 and H3 were raised against gp120 envelope protein^35^, H4 against gp41^40^ and H5 against gp140 protein^37^. Hep1 binds the hepatitis B virus (HBV) surface antigen (HBs)^46^. This HBs is expressed in HBV virus like particles (ADW & ADY). Bispecific VHHs generated in this study are H2E2, E2H2, H2E1, H3E1, H4E2, E2H4, H5E2, Hep1E1 and control biheads E2E1, H2H2, H5H4.

**Figure 1:**
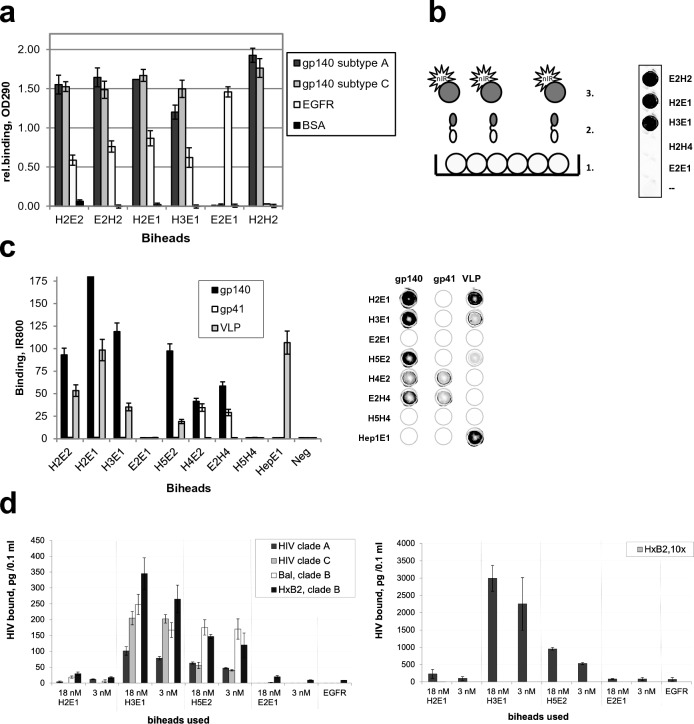
Bispecific VHH llama antibodies target HIV to EGFR ectodomain. (**a**) The VHHs in bispecific constructs retain good binding properties. Wells were coated with antigen, binding measured by ELISA. n = 2, representative experiment shown. (**b**) Bispecific VHHs mediate binding of HIV- antigens to EGFR ectodomain. Schematic set-up [with 1 = immobilised EGFR in wells, 2 = (heterospecific) bihead VHHs and 3 = IR800 labeled HIV-1 protein] and IR800-labeled gp140 UG membrane protein specifically targeted to EGFR in wells. n > 2, representative experiment shown. (**c**) Overview of relative efficacies of targeting by bispecific VHHs. gp140 is gp140(UG37), Virus like particle (VLP) is VLP_HIV,_ except for Hep1E1 it is VLP_HBV._ Shown are quantitative IR800 FLISA results and for overview an IR800 scan of a representative microtiter plate (dotted circles mark the wells) is shown. n = 2. (**d**) Efficient targeting of low and high concentration HIV-1 to the coated EGFR ectodomain. Viruses were pre-incubated with 18 nM or 3 nM biheads for 1 h and bound to EGFR coated ELISA plate for 2 h. Bound HIV was detected by a p24 ELISA. *Left graph* Viral concentration in the input was 10.1, 4.9, 3.9 and 9.0 ng/100 µl for clade A (92UG037), clade C (96ZM651), and clade B reference strains Bal and HXB2, respectively. *Right graph* The same with 10 times concentrated HXB2. Clade b, n = 2, mean ± sd; Clade A and C, n = 1, duplo shown.

After fusion into a bihead, both the anti-HIV-1 and the anti-EGFR VHHs retain excellent binding properties, independent of the orientation of VHH antibody moieties in these molecules. This was demonstrated in ELISA analyses showing binding on purified EGFR ectodomain protein and on purified HIV-1 envelope proteins (clade A, 92UG037, and clade C, gp140ZM96) (Fig. 1a). Control proteins E2E1 and H2H2 demonstrated specificity of the VHHs as no binding was observed with these monospecific biheads to the other target. Next, we investigated whether the bispecific VHHs can recruit HIV-1 proteins to immobilized EGFR ectodomain. Instead of using multistep ELISA methods, we covalently labeled the HIV-1 proteins with the near infrared dye IR800CW, which allowed us to obtain quantitative binding results in IR800 FLISA (fluorescence linked immunosorbent assay). Using a scanner, bound IR800-labeled HIV-1 protein is directly detected in the wells. Clearly, unlike H2H4 and E2E1 controls, the bispecific VHHs mediate recruitment of the IR800-labeled envelope proteins to coated EGFR (Fig. 1b). We next expanded our panel of bispecific VHHs including VHHs binding to gp41. Functional envelope glycoproteins at the surface of viruses consist of trimers of covalently coupled gp41 and gp120 proteins, of which gp41 has a transmembrane region. In this study we used trimeric gp140 molecules that contain gp120 plus the ectodomain of gp41, stabilized by the addition of heterologous trimerization motifs at the C-terminus of the gp41 sequence. H2 and H3 bind to a gp120 epitope only and in accordance do not recognize gp41 trimers. H4 (α-gp41) binds the gp41 part of gp140 and H5 binds gp140^37^ (Fig. 1c).

The recombinant glycoproteins gp41 and gp140 display a native conformation and form trimers. However, as the real HIV-1 virus particles are much larger and have complex membrane structures, we produced non-infectious HIV-1 virus-like particles (VLPs) that have the size of virions and express the ZM96 strain (clade C) envelope proteins in their membrane^45^. In addition, to show that directed VHH-mediated recruitment is not limited to HIV-1 but may be more generally applicable, we also tested hepatitis B virus like particles using Hep, a VHH specific for this type of virus^46^. The HIV- and hepatitis B VLPs were labelled with IR800 dye and are also shown to be specifically targeted to EGFR depending on the bispecific VHH used (Fig. 1c). Exceptions are H4E2 and E2H4 that target gp41 to EGFR, but not HIV-1 VLP, probably being too short for linking or due to other steric hindrance. Under the labeling conditions use, there were never any indications that labeling interfered with binding. In conclusion, we generated bispecific VHHs that can specifically target viral envelope glycoproteins as well as HIV-1 and HBV virus-like particles to EGFR.

Note that at modest to low antibody concentrations (nM) the VLP (pM) bind well to EGFR. The trimers contain three binding sites which will contribute to a higher avidity due to cooperative actions of multiple binding. This is illustrated for gp140 trimers by quantitative FLISA experiments (Supplemental Fig. S1a). Due to a large number of viral membrane proteins per particle, the VLPs contain even more binding sites, resulting in high avidity. Consequently, in competition experiments the preincubated VLPs, containing many binding groups at the surface of the particle were only twofold inhibited upon addition of 20-fold higher concentrations of competing free antibodies (Supplementary Fig. S1b)*.* It illustrates the power of binding avidity when multiple epitopes per particle are involved.

To test infectious HIV-1, two common HIV-1 reference strains HxB2 (clade B) and BaL (clade B) and two patient derived HIV-1 strains 92UG037 (clade A) and 96ZM651 (clade C), were cultured and tested for bispecific VHH mediated targeting to the EGFR ectodomain (Fig. 1d). Dependent on their binding efficiency for different clades the bispecific VHHs were shown to be effective and concentrations of 3 nM were sufficient to bind envelope proteins and target all four infectious HIV-1 strains to the immobilized EGFR. Since 3 nM of bispecific VHHs already provides a molar excess over HIV-1 envelope proteins, increasing the concentration to 18 nM hardly increased the amount of HIV-1 that is bound to EGFR. With a 10 × increase in HIV-1 added, on average 8 × more HIV-1 was targeted to EGFR (Fig. 1d), indicating the potency of the bispecific VHHs. In conclusion, bispecific VHHs can efficiently recruit HIV-1 reference strains and clinical isolates to EGFR ectodomain in vitro.

### Targeted internalization and degradation

**Figure 2:**
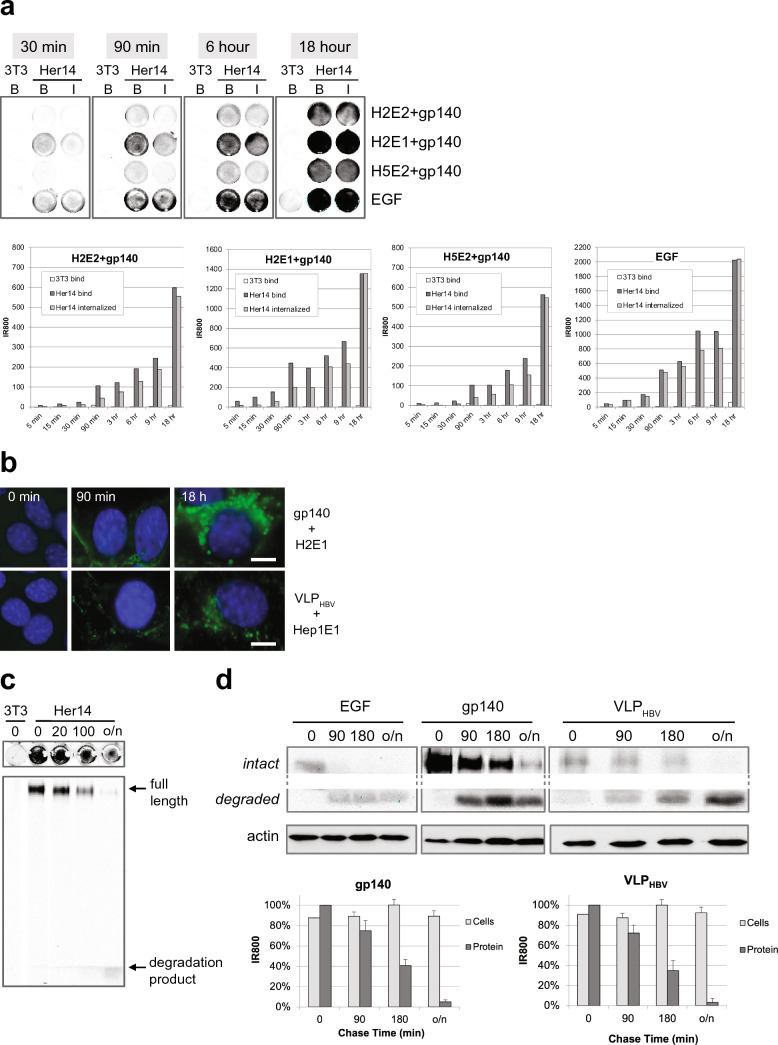
Binding, internalization and degradation in EGFR expressing cells. (**a**) Kinetics of binding and internalization. Labeled antigen (gp140 UG37) was pre-incubated for 30 min with bispecific VHHs and incubated with cells. At indicated times, cells were washed showing the total bound antigens (wells B) or stripped to remove antigens at the cell surface showing the internalized antigens (wells I). Bound (B) and internalized (I) antigens were scanned and quantified for IR800. Upper panel is a IR800 scan of some wells for illustration. Lower panels, graphs for quantification. The difference between bound and internalized represents the amount that is still exposed to the surface of the cells. All four display similar kinetics of net binding, whereas internalization is more variable. n = 2, representative experiment shown. (**b**) Immune fluorescence shows that envelope proteins gp140 and Virus Like Particles internalize similarly. Covalently green fluorescently labeled antigen (gp140, VLP_HBV_) was pre-incubated with bispecific VHHs and added to cells for 0, 90 min and 18 h. Blue are nuclei, scale bar: 5 µm. n = 2, representative experiment shown. (**c**) Bispecific VHH mediated internalization results in rapid degradation of HIV proteins in the cells**.** Development of IR800 based internalization and degradation assay. *Top:*. Preincubated complexes IR800-labeled gp140 plus bispecific VHH H2E1 bound for 2 h on ice to the indicated cells in 48-wells. After washing, chasing occurred in conditioned medium at 37 °C for the indicated time, in min. Cells were washed and scanned for IR800. *Lower.* The cells in the wells were next solubilized and proteins were separated and analyzed on a SDS-PAGE gel for IR800. Note that after binding (0 min chase) only full length HIV envelope protein is observed; after overnight chasing (o/n) mostly labeled protein degradation products are detected, in the front of the gel. n > 2, representative experiment shown. (**d**) Comparison of the kinetics of degradation of IR800 labeled EGF, gp140 and VLP_ADW_. After labeling gp140 and VLP_HBV_ were firstly preincubated with bispecific VHH H2E1 and Hep1E1, respectively. Next incubation was on Her14 cells on ice for 2 h, cells were washed and complexes internalized for 0 min, 90 min, 180 min or overnight at 37 °C. Subsequently cells were lysed, run on SDS-page gel and scanned for IR800. Next, the gel was blotted to PVDF to perform Western blot analyses with α-actin, to confirm equal loading. IR800 scan of the gels shows the IR800 labeled EGF, gp140 and VLP_ADW_, resp. Intact protein and degraded protein are part of the same gel. Actin panel is a Western blot. n = 2, representative experiment shown. *graphs*: IR800 quantification of intact proteins and total IR800 dye in the cells, n = 2, mean ± sd.

Next, we aimed to study bispecific VHH mediated binding of HIV-1 antigens to cells expressing EGFR, their internalization and the intracellular fate of the internalized viral proteins. Viral antigens gp140 and VLPs were labelled with IR800 and pre-incubated with bispecific VHH for 30 min. The resulting preformed antigen–antibody complexes were added to Her14 mouse fibroblasts cells expressing the EGFR receptor and to NIH3T3 2.2 cells that lack expression of EGFR. As a positive control for the experiment, we took EGF labelled with IR800. After binding for the indicated time points (Fig. 2a), cells were either washed to determine bound gp140-IR800 (panels B, bound) or they were acid stripped to determine internalized HIV envelope protein (panels I, internalized). As shown in Fig. 2a, we found that antigen–antibody complexes bound specifically to EGFR in Her14 mouse fibroblasts cells and not to negative control cells NIH3T3 2.2. Binding as well as the internalization of HIV proteins increased continuously over 18 h. As expected, the positive control EGF-IR800 showed rapid internalization (compare the differences between internalized and bound) as compared with the internalization of HIV-1 envelope proteins. This is due to the fact that EGF-IR800 activates the EGFR receptor resulting in rapid internalization. In contrast, our bispecific VHH antibodies do not activate the EGFR^41,42^. Nevertheless, we still observed continuous uptake of the bound envelope proteins increasing from 35%, 80%, to 95% after 90 min, 6 h and 18 h, respectively. Unfortunately, the membrane glycoprotein of HIV binds to proteoglycans^47^ which are extracellular matrix components that are produced by many cell types, particularly fibroblasts. Indeed infective HIV-1 as well as HIV-1 VLP bound non-specifically also to control cells lacking EGFR (Supplemental data Table S1). However, we found that Hepatitis B VLP (VLP_HBV_) do not bind non-specifically and provide therefore a good model to investigate internalization and fate of particles with the size of viruses, VLPs, in tissue culture. Gp140 and Hepatitis B VLPs were covalently labeled with AlexaFluor488 and internalization was demonstrated by immune fluorescence microscopy (Fig. 2b). After 90 min incubation, labeled proteins were observed in endocytosed vesicles for both antigens. After overnight incubation all signal was present in endocytosed, mostly perinuclear, vesicles. Thus, we conclude that even without EGFR activation, bispecific VHH mediated targeting results in clear uptake of viral envelope proteins and VLPs by EGFR-expressing cells.

### Bispecific VHH-mediated uptake results in lysosomal degradation

Binding of the natural ligand EGF to the receptor EGFR activates a negative feedback loop resulting in internalization and degradation of receptor-growth factor complex^48–50^. We assessed whether the bispecific VHH mediated uptake of the virus would also subsequently result in degradation of the complex components, even without EGF growth factor activation. Degradation was analyzed using covalently IR800 labeled EGF, gp140 and VLP_HBV_ proteins. Of note, we found that IR800 dye resides inside cells, even upon degradation of the carrier protein it is conjugated to. This allows for straightforward quantification of protein-linked and protein free dye in cell lysates. Cells were incubated with IR800-labeled proteins for various periods. After quantification of IR800 in the FLISA wells, we solubilized the cells and analyzed the quantity and intactness of the proteins by denaturing protein gel analyses, using quantitative analysis of IR800. Figure 2c shows that upon internalization of recruited HIV-1 envelope proteins (as in Fig. 2a), they become rapidly degraded (decrease of the full length gp140 signal). At 100 min of chase most dye is still present in (or on) the cells, however over 40% of the (internalized) envelope proteins are degraded and some IR800 labeled degradation products are visible on the gel. Supplementary Figure S2 shows similar results with the other bispecific VHHs, The degradation kinetics of recruited gp140 were compared with that of VLPs and the EGFR ligand EGF (Fig. 2d). As expected EGF is degraded more rapidly, degradation of both the HIV-1 proteins and VLPs is slower. However degradation was almost complete within a day. Western blot analyses measuring actin confirms that equal amounts of cell lysates were loaded in these experiments. Binding and degradation were similar when tested in other EGFR expressing cell types, including Hela, A431, and 14C cells (Supplementary Fig. S3).

**Figure 3:**
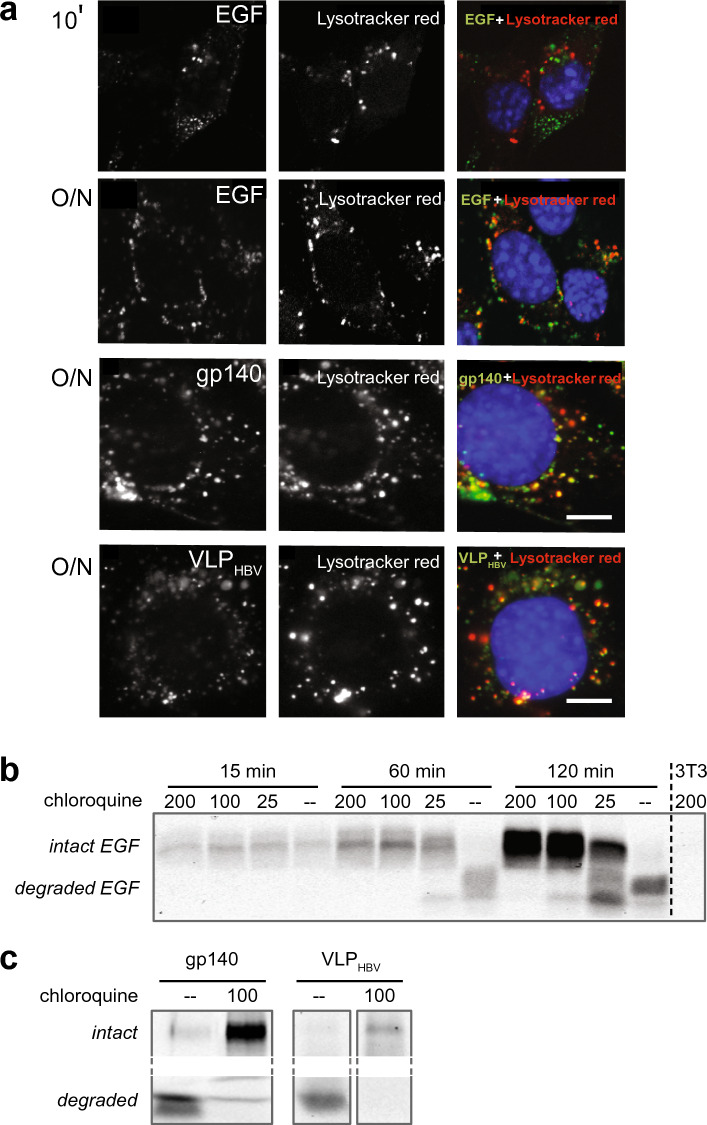
Bispecific VHH mediated recruitment results in lysosomal degradation. (**a**) Colocalization of Alexa-488 fluorescently labeled EGF, gp140 and VLP (green) with lysotracker red in EGFR expressing cells. EGF was bound for 10 min and overnight, viral proteins were internalized overnight, mediated by bispecific VHHs H2E1 and Hep1E1 resp. Nuclei are blue, scale bar: 5 µm. n = 2, representative experiment shown. (**b**) Chloroquine inhibits EGF degradation. SDS-PAGE gel of IR800 labelled EGF in cells. EGF was incubated on cells after 60 min pretreatment with chloroquine (µM indicated). Cells were lysed, run on SDS-PAGE gel and IR800 was detected. n = 2, representative experiment shown. (**c**) Similarly, chloroquine inhibits gp140 and VLP_HBV_ degradation. SDS-PAGE gel of IR800 labelled gp140 and VLP_HBV_ internalized in cells after treatment with or without chloroquine (100 μM). Intact and degraded are in the same lane in the gel. n = 2, representative experiment shown.

Next, we investigated the observed bispecific VHH mediated degradation of viral proteins in the EGFR expressing cells. Upon EGF binding to the EGFR, the complex is internalized, sorted to early endosomes, late endosomes and finally degraded in the lysosomal compartment. Therefore, mouse fibroblasts cells Her14 were labelled with lysotracker dye that stains acidic compartments like lysosomes. After internalization of labeled proteins (fluorescently labelled EGF, gp140 and VLP) mediated by bispecific VHH, the colocalization of the protein and lysotracker was analyzed with fluorescently labelled EGF, gp140 and VLP. Whereas Fig. 2a showed that at 30 min incubation most of the bound EGF is internalized, Fig. 3a shows that it is present in small endocytotic vesicles without co-localization with lysosomes. After 30 min colocalization occurs increasingly and it is completed in 3 h (data not shown), which is maintained at least up to 18 h (Fig. 3a, overnight panel [O/N]). After overnight incubation, fluorescent (degradation) products of EGF, recruited HIV-1 envelope proteins and VLPs show similar lysosomal localization. This result suggests that targeting mediated by bispecific anti-HIV-1 VHH for viral antigens to EGFR expressing cells leads to internalization and degradation by the lysosomal degradation pathway.

To further confirm these observations, mouse fibroblasts cells Her14 expressing EGFR receptor and negative control NIH3T3 2.2 cells were incubated with the lysosomal inhibitor chloroquine^51^. The most appropriate functional range of concentration of chloroquine to inhibit lysosomal degradation in these cells was established with EGF-IR800, at chloroquine concentrations 0, 25, 100 and 200 µM (Fig. 3b). Chloroquine inhibits EGF degradation dose-dependently and is already optimal at 100 µM concentration. In cells not treated with chloroquine, internalized EGF is still intact after 15 min but completely degraded after 60 min. Similarly, Fig. 3c shows IR800 labeled envelope protein (gp140) and IR800 labeled hepatitis virus like particles (VLP_HBV_), that were preincubated with the corresponding bispecific antibodies and incubated overnight on cells. Without chloroquine (–) they are largely degraded whereas 100 µM chloroquine prevented degradation, indicating that after uptake these proteins are targeted for lysosomal degradation. Further analysis of EGFR signaling pathways indicates that the internalization does not per se require EGFR activation and is not accompanied by activation of signaling pathways associated with tumorigenic signaling (Supplemental Fig. S4).

### Bispecific VHH-mediated lysosomal degradation of infectious HIV by EGFR expressing cells

Next, the infectious HIV-1 reference strain HxB2 was analysed for targeted degradation in cells. Like many viruses HIV binds to heparin sulphate proteoglycans and thus also to the fibroblast cells. In vivo this “nonspecific binding” is thought to play a physiological role in infection by pre-concentrating the virion particles at the cell surface, as an intermediate step towards specific binding to CD4^47^. To facilitate determining the specific effects of the biheads in our study, we firstly investigated methods for blocking non-specific binding using IR800 labelled HIV-VLP. None of these gave a satisfactory result, but bispecific VHHs containing an anti-HIV and an anti-EGFR moiety appeared to mediate on average 30% more binding than control biheads that contain two anti-HIV-1 moieties (Supplementary Table S1). Thus, for the VLPs the bispecific VHHs yield a modestly higher binding than background binding alone.

**Figure 4:**
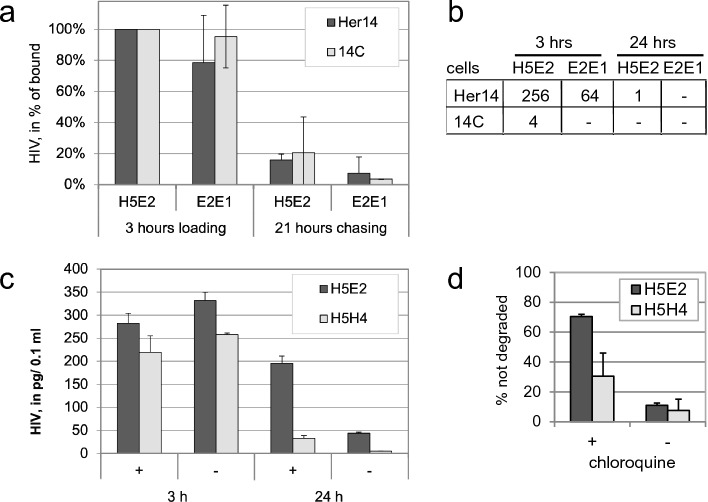
Specific binding of infective HIV particles/virions to EGFR expressing cells results in internalization and lysosomal degradation. (**a**) HIV bound to cells is internalized and degraded upon incubation**.** Pre-incubated HXB2 was incubated with Her14 and 14C cells (both expressing EGFR) for 3 h (loading), and non-bound virus washed away. Viral protein was determined directly (loading) and after 21 h, by measuring p24 in cell lysates by ELISA. n = 2, mean ± sd. (**b**) Trans-infectivity assay. Separately, after the indicated time points MT2 indicator cells were added to establish trans-infectivity of the bound HIV. Titre indicates the viral dilution at which syncytia are still formed. n = 2, identical results. (**c**) Chloroquine inhibits degradation of internalized HIV. Her14 cells were treated ( +) or not (-) with 100 µM chloroquine and incubated with virus for 3 h, in the presence of heparin Then washed and incubated for 24 h. HIV presence was determined by p24 ELISA. n = 2, representative experiment shown. (**d**) The graph indicates the percentage of HIV that is not degraded after 24 h as percentage of the input that is bound after 3 h. n = 2, mean ± sd.

Subsequently, infectious HIV-1 HxB2 virus particles were incubated with bispecific VHH for 1 h, to form HIV-antibody complexes. These complexes were then added to the wells containing either Her14 or 14C cells (both expressing EGFR), for 3 h (loading). Then, non-bound virus was removed by washing and the cells were left for 21 h (chase). Viral protein was detected in lysates from treated cells using HIV-1 p24 ELISA. The amount of HIV-1 on or in the cells present after 3 h binding (loading) decreased by 82–96% after 21 h chasing (Fig. 4a), both with bispecific VHH antibody and negative control VHH antibody, due to release of the bound virus during the chase or due to internalisation and degradation. Whereas this was non-discriminative, trans-infectivity assays performed in parallel indicated that binding by bispecific VHH is 4 times more effective than controls in presenting HIV-1 to MT2 indicator cells (Fig. 4b). Virus was bound for three hours, washed and cocultured with MT2 indicator cells for 7 days after which HIV-1 mediated syncytia were scored. This was performed with subsequent dilutions of the treated viruses and the highest dilution at which syncytia are observed is shown (Fig. 4b). Effectiveness of H5E2 was most clearly shown with Her14 cells. The 14C cells bound HIV as efficiently (Fig. 4a) but apparently present the virus in a less functional way for infection of the indicator cells to occur ór the viruses are internalised so rapidly that they cannot infect the indicator cells anymore. After 21 h chasing, infectious virus was no longer present at the surface of Her14 cells, suggesting that after initial loading/binding the virus is internalized and degraded. To test if degradation occurs in lysosomes, the cells were treated with chloroquine that inhibits lysosomal degradation whereas it does not influence internalization (Fig. 4c). We show upon 3 h binding with and without chloroquine and washing that HIV-1 is bound, with about 20% more binding with the H5E2 than with the control bihead H5H4. Subsequently, about 90% of the non-specifically bound virus (H5H4-treated) is lost during chasing for 21 h, both in the presence and absence of chloroquine. With H5E2 in the presence of chloroquine 70% of the virus is still intact in the cells. This implies that despite a high level of nonspecific binding 70% of the H5E2 treated viruses cells are internalized and degraded in lysosomes, as can be inhibited by chloroquine. Apparently, despite nonspecific binding the H5E2 targets the virus into the lysosomal degradation pathway. The graph in Fig. 4d indicates the percentage of HIV that was present after 24 h as percentage of the input that is bound after 3 h. Thus, most of the bound HIV-1 in the presence of bispecific VHH is mediated to lysosomal degradation that is inhibited by chloroquine. whereas the non-specifically bound HIV was released or, hypothetically, may follow a non-EGFR dependent internalization and degradation route. In conclusion, the bispecific VHHs mediate targeting of infectious HIV to the lysosomal degradation pathway resulting in efficient degradation.

## Discussion and conclusion

In this study we make use of various strong properties of the VHH antibody technology. Llamas are immunized with (cocktails of) the desired antigens and this resulted in a large number of VHH antibodies, retrieved from constructed phage libraries^33–37^. Phage display and the use of clever selection methods allows recovery of VHH antibodies with desired properties. Thus, previously a large number of VHHs were selected that recognize different epitopes on HIV-1 ensuring the coverage of a very broad range of different clades of HIV-1^35^ as well as VHHs recognizing different epitopes on EGFR^41,42^. This allowed us to pursue a novel strategy to clear HIV-1, of which we show proof of principle in the present study. Whereas the immune system has a remarkable adaptive efficacy to fight unwanted agents in the body, pathogenic viruses like HIV have evolved such that they are capable to escape from natural clearing mechanisms^52^. Our approach is to circumvent this, by targeting the virus for degradation by cells in the body to which the virus is not adapted. We provide proof of principle for this method using bispecific antibodies composed of single domain llama antibodies [VHHs] to capture HIV-1 and target it to cells expressing EGFR, resulting in endocytosis and lysosomal degradation.

EGFR internalization has been studied for decades, mostly related to EGF signaling, endocytosis, regulation of receptor degradation and recycling^48–50^. Here we focus on the fate of the bound molecules, EGF-IR800, viral proteins and HIV viruses. Quantitative detection of IR800 in cells and gels demonstrated targeting to degradation using ”pulse chase like experiments” (bind, wash and follow intactness in time, Figs. 2 and 3). As expected, growth factor EGF rapidly activates EGFR endocytosis and is degraded within 90 min. whereas the bispecific VHH mediated degradation takes 24 h (Fig. 2d). Our working hypothesis is that the bispecific VHH mediated complex is piggybacking the natural turnover of the EGFR, no EGF is required for this process. Interestingly, in the continuous presence of the reagents the net internalisation mediated by bispecific VHHs shows comparable kinetics as EGF internalisation (Fig. 1a). Similarly, in vivo too the amount of the (ubiquitously expressed) EGFR will determine how much virus will be bound and degraded in a certain period of time.

It is difficult to completely rule out that the forced internalization of HIV in cells might have the adverse effect that some infection occurs via this route. In general however, since earlier controverses about the fate of HIV-1 in endosomal vesicles, much evidence indicated that HIV-1 is degraded in lysosomes^53–55^ and generally requires CD4 and a coreceptor for productive infection^56–58^. In addition, not all cell types will provide all the gene products required to support viral propagation^59–61^. Note that also the natural Fc-mediated degradation of HIV-1 bound by conventional antibodies was recently indicated to occur by endocytosis (into liver sinusoidal endothelial cells) and subsequent degradation in lysosomes^62^. Nevertheless, future in vivo experiments will have to provide more definite proof to address this issue.

We consider several putative advantages of our method. Conventional antibodies bind to HIV through two variable domains and require engagement of the immune system through their constant domains (Fc), before finally the viruses are cleared. This takes time and is most likely the reason that neutralizing antibodies are required^8,63^. EGFR is ubiquitously expressed on many (non-immune) cells in the body. It will be interesting to see if viral clearance by these bispecific VHHs might be so efficient and rapid in vivo that only binding, without the need for neutralization, will be sufficient. Many neutralising VHHs are available against HIV^33,35,37^ and for certainty we have also used 2E7 = H5 in this study that binds and neutralises most strains belonging to the A, A/G, B, B/C and C subtypes^37^. However, if future studies will show that neutralizing antibodies are not required using our approach, that will save much time in selecting the best.

Single antibody treatments of viremic individuals have resulted in the development of resistant HIV-1 strains. However, combination of various antibodies significantly reduced this^6,9–12,64^. The use of BsAbs, binding 2 different epitopes on HIV-1^27^ or binding HIV together with a HIV-1 receptor molecule^28^ may give further improvement but required extensive antibody engineering. VHH technology has the advantage that it provides a framework in which many functionalities are easily coupled as modular building blocks^26,31,32,37^. Also, similarly, combinations of bispecific VHHs with VHH domains that recognize different epitopes on HIV-1 envelope proteins may be used to prevent mutational escape by the virus. The bispecific VHHs are 5 times smaller than conventional antibodies and thus with the same amount of protein injected, at similar molar concentrations combinations of a larger number of different bispecific VHH antibodies can be administered. Moreover, in our experiments the bispecific clearing VHHs were shown to function at low concentrations (nM) and with VLPs even at pM concentrations. This is less than the concentrations of neutralizing anti-HIV-1 mono-head VHH that are required for neutralization^35^. This difference is likely to be related to a difference in mode of action: neutralization may require binding to all individual envelope proteins on the virus whereas in contrast a few bound bispecific VHHs per virus particle might suffice to target the virus to EGFR expressing cells, leading to its clearance. Thus, probably much lower concentrations of antibodies will be required using our method of clearing HIV.

In general, binding interactions of viruses with antibodies are limited due to a very low molar concentration of virus particles but on the other hand each virus particle has about 15 trimeric envelope proteins on their surface^65^ resulting in a large avidity effect. Our experiments indicate that even at low concentrations of bispecific antibodies this results in a sufficient high percentage of HIV-1 particles being decorated with the bispecific VHHs and therefore recruitment to cells that display EGFR on their surface. When injected into the blood the residence time of the bihead in the circulation is less than that of conventional antibodies. Whereas this might be a disadvantage, VHH technology has several ways to prolong the half-life of these molecules^31^. For example, by employing an anti-albumin VHH we previously showed the tunability of VHH residence time in vivo^66^. Moreover, variants of anti EGFR VHHs are already used extensively in clinical trials concerning breast cancer therapy, from which much physiological and practical information can be derived. Moreover, nanobodies (VHHs) developed by Ablynx are approved by the FDA^67^.

Thus, the bispecific VHHs may present a versatile alternative for the conventional (bispecific) antibodies, with potentially efficient and rapid clearance of HIV in the circulation. Since VHHs are stable and functional in vaginal environments^68^ and effective as shown in a small trial with macaques (CT Verrips et al. accepted for publication), our approach may also be suited for prevention when used in microbicides. Viruses may be captured by the bispecific VHH before coming into contact with their natural target cells. When passaging the tissue lining the bispecific VHH would target the virus to epithelial cells that internalize and degrade it. Also due to simple cloning methods it is easy to substitute the anti-HIV-1 and anti-EGFR antibody domains for other specificities. Currently, we are testing whether this platform can be used to eliminate other viruses and toxins. Of course, it will be especially interesting to see if SARS-CoV-2, for which VHH antibody domains have also been selected^69–71^, can be tackled with our method.

## Methods

### Construction of bihead VHHs and production

The anti-HIV VHHs 1F10 (H2), 2E7 (H5)^37^, 2H10 (H4)^40^, H3 (this study, sequence = EVQLVESGGGLVQPGGSLRLSCAASGSILDDANAMGWYRQTPGTERALVALITDSGATRYADSVKGRFTISRDNAKNTATLQMNSLKPEDTAVYYCNFREFGGWGTNIDHWGQGTQVTVSS) and the anti-EGFR VHHs EGa1 (E1) and EGb4 (E2) (Hofman)^41^ were combined in biheads in various combinations using standard PCR techniques, with a linker encoding GGGGSGGGGS. The N-terminal VHHs were cloned using a 5’ primer containing a *PstI* digestion site (5’ GTTCCATTCTATGCGGCCCAGCCGGCC) and a 3’ primer encoding part of the 10 amino acid (AA) linker, including a *BamHI* digestion site (5’TCAGTAACCTGGATCCCCCGCCACCGCTGCCTCCACCGCCTGAGGAGACGGTGACCTG). The C-terminal VHH was amplified using a 5’ primer containing the second part of the linker including a *BamHI* digestion site (5’ AGGTTACTGAGGATCCGAGGTGCAGCTGGTGGAGTCTGG), while the 3’ primer contained a *NotI* digestion site (5’GGGACCCAGGTCACCGTCTCCTCA). PCR fragments were digested with *PstI, BamHI* and *NotI* (Fermentas), agarose gel purified, and cloned together into a phagemid vector for display on filamentous bacteriophage digested with *PstI* and *NotI*. Resultant clones contain a Myc-tag and a His tag. Expression was in *E.coli* TG1, DH10 or DH5α. Colonies were screened for insert by colony PCR using M13 primers. Expression of recombinant VHH proteins in *E. coli* and purification by immobilized metal ion affinity chromatography (IMAC) were performed with His tag binding Talon-beads (Clontech) as described^34^. The isolated product  was checked for purity on a Coomassie-Blue-stained 15% SDS-polyacrylamid. All clones were confirmed by DNA sequencing.

### Covalent labeling of proteins and VLP with fluorescent dyes

HIV envelope protein gp140(UG37), subtype A, was kindly provided by Dr S. Jeffs, Wright-Fleming Institute, Division of Medicine, Imperial College London, London. Recombinant HIV-1 envelope protein gp140CN54, subtype C, was obtained from the Centre for AIDS Reagents, NIBSC HPA UK, supported by the EC FP6/7 Europrise Network of Excellence, and NGIN consortia and the Bill and Melinda Gates GHRC-CAVD Project and was donated by Polymun, Immunodiagnostics, Immune Terchnology. gp41(GCN) contains extracellulair domains linked together to form a trimer^40^. The HIV protein based VLP’s (VLP_HIV_) presenting the envelope protein ZM96 gp145, subtype C, were generated in 293 T cells after large scale transient co-transfection with 2 plasmids encoding HXB2 Gag and ZM96 gp145 comprising the complete external gp120 moiety, the extracellular domain of gp41 as well as its transmembrane domain. VLPs harboring the HIV envelope proteins were sucrose gradient purified essentially as described^45^. Hepatitis B virus like particles (recombinant surface antigen ADW subtype HC87-2) were obtained from HyTest Ltd. For labeling proteins concentrations were ≥ 1 mg/ml. If necessary, proteins were concentrated by Amicon® Ultra Centrifugal Filters or by Microcon YM-3 spin- columns (Millipore).

VHH and other proteins were labeled with a ratio of 20 µg: 0.67 µg IRdye800 (IRdye 800CW NHS ester infrared dye from Licor, product 92970020) in PBS at room temperature, shaking, for half an hour. For the VLPs this was 20 µg with 2 µg dye and 1/10 vol 0.5 M NaHCO3 pH 9. Reactions were quenched with 10% of 2-(Methyl-Amino) ethanol (1 M, pH 9.0, Sigma), and unbound dye was removed from the labeled protein by size exclusion separation over homemade 1 ml G-25 Sephadex (GE Healthcare) column. Protein concentration and labeling efficiency were determined with a Nanodrop 1000 spectrophotometer (Thermo scientific). For immune fluorescence proteins were labeled similarly with Alexa 488-NHS (Invitrogen).

### ELISA

96-well MaxiSorp plates were coated overnight with gp140 subtype A (= UG37), and subtype C (= CN54) or BSA control (250 ng /50 µl PBS). After blocking with 200 µl 4% w/v skimmed milk (Marvel, in PBS). VHHs were added for 1 h (200 ng/100 µl). Incubations were in 1%Marvel in PBS, all washings with PBS with 0.05%Tween-20. Detection was with αMyc (Roche Diagnostics, 1/2000) or αHis (Amersham, 1/5000), peroxidase-conjugated secondary antibodies (Jackson Immunoresearch, 1:5000) and o-Phenylenediamine (OPD). Secondary antibodies used were donkey anti-mouse and donkey anti-rabbit IgG.

### EGFR binding of biheads, FLISA and ELISA

Coating was with polyclonal Rabbit anti-Human IgG (DakoCytomation, 1/2000 in 50 µl PBS). After blocking with skimmed milk (4% w/v Marvel in PBS), incubation was with EGFR ectodomain (EGFR-ect) containing an Fc-tail (85 ng in 50 µl 2% Marvel) and next incubated with biheads at indicated concentrations in 1% Marvel. In FLISA results were analyzed by the Odyssey Infrared Imaging System (Li-Cor Biosciences). For ELISA αMyc (1/2000), αMouse-Ig-peroxidase (1/5000) and *o*- Phenylenediamine (OPD) were used.

### Preincubated complexes

IR800 labeled proteins and VLP were preincubated with 2 to threefold molar excess biheads to form preincubated complexes for 30 min at room temp. (typically 50 ng gp140-IR800 or VLP-IR800 + 37 ng bihead VHH in 20 µl). For ELISA and FLISA preincubation was in 2% Marvel, for application on cells in 1% BSA (filter sterilized). Binding of preincubated complexes in assays was typically with 5 ng labeled protein in 200 µl i.e. 2 nM preincubated complex.

### Bihead mediated HIV-1 binding

The HIV-1 strains used were propagated and purified by standard procedures and resulting concentrations were determined by HIV-1 p24Ag ELISA (Aalto Bioreagents): HXB2, 940 ng/ml p24Ag; Bal, 109 ng/ml; 92UG037 (NIH Aids Reagent Program), 37 ng/ml; 96ZM651 (NIH Aids Reagent Program), 24 ng/ml p24Ag. Required amount of virus was spun for 1 h, 17,000 rpm at 4 °C and the virus pellet was resuspended in PBS + 2% BSA. 100 µl (typically 10 ng viral p24) was incubated with 60 ng or 10 ng bispecific bihead (resp. 18 nM and 3 nM bihead) for 1 h 37 °C for preincubation. Virus- bihead complexes were incubated in EGFR coated wells for 2 h. 37 °C, washed 4 times with PBS and bound virus lysed in 100 µl 0.1% empigen (Sigma) in TBS which was transferred and analyzed by p24 ELISA.

### Cell culture

The murine fibroblast cell lines NIH 3T3 clone 2.2 (indicated 3T3) lacking EGFR expression and HER14 is derived from it as a stable transfectant expressing human EGFR. The tumor cell line UM-SCC-14C was kindly provided by G.A.M.S. van Dongen (Department of Otolaryngology, VU University Medical Center, Amsterdam, The Netherlands). Cells were cultured in Dulbecco's modified Eagle's medium (DMEM) supplemented with 7.5% fetal bovine serum (v/v), 100 U/ml penicillin, 100 µg/ml streptomycin and 2 mM L-glutamine (all Gibco, Invitrogen) at 37 °C in a 5% CO_2_ humidified atmosphere. For incubations outside the incubator the medium was replaced by CO_2_ independent medium (Gibco, Invitrogen).

### Internalization assays

Corning plate wells were coated with 0.25% gelatin (Merck, autoclaved) and washed with PBS. Her14 and 3T3 2.2 cells were seeded 4 × 10^4^ cells/well in a 48 wells plate and grown overnight in DMEM medium at 37 °C in a CO_2_ containing environment. EGF-IR800 (LiCor) or preincubated complexes were added to the medium and incubated for 30 min, 90 min, 6 h or 18 h. Cells were washed twice with CO_2_ independent medium, cooled on ice and stripped once for 5 min with strip buffer (250 mM NaCl, 100 mM Glycine, pH 2.5). Cells were washed twice with CO_2_ independent medium and IR800 measured by Odyssey.

### Fluorescence microscopy

Cover slips (Menzel-Gläzer) in a 24 wells plate were coated with 0.25% gelatin and washed with PBS. 2 × 10^5^ Her14 cells or 3T3 2.2 cells per well were seeded on the cover slips and grown overnight. Cells were incubated with Alexa488 labelled EGF (Life technologies, 8 nM), or homemade Alexa488 labelled VLP_HBV_ (200 ng) and gp140UG37 (2 nM) that were preincubated with biheads. After PBS washing cells were fixed with 4% Paraformaldehyde (PFA) (Sigma) in the dark for 30 min at RT. Subsequently, cells were washed and treated with 20 ng/ml 4',6-diamidino-2-phenylindole (DAPI, Roche Diagnostics Corporation) and embedded in Mowiol (Sigma) with PPD on a microscope slide. For colocalization with lysotracker cells were incubated with EGF-Alexa488 for 10 min or with preincubated complexes of bispecific VHH H2E1 with gp140-Alexa488 and Hep1E1 with VLP_HBV_-Alexa488 for 6 h, after which cells were washed 3 times with PBS and grown in fresh DMEM (8% FBS). After o/n growth 90 min before washing and paraformaldehyde fixing lysotracker red (Life technologies, 75 nM), was added to the cells. Images were obtained using a Zeiss Axiovert 200 M confocal microscope (Carl Zeiss Microscopy GmbH, Germany) equipped with a 63 × water-immersion objective (NA 1.2).

### Degradation assay

Wells were coated with 0.25% gelatin. Her14 and 3T3 2.2 cells were seeded 4 × 10^4^ cells/400 µl/well in a 48-wells plate and grown overnight. Cells were cooled on ice and medium was collected as conditioned medium and replaced by ice cold CO2-independent medium containing 0.5% BSA (Sigma) and 0.4% FBS (300 µl/well). After washing once, EGF-IR800, bihead VHH or preincubated complexes were added (usually in 50 µl to mix rapidly) and incubated on the cells for two hours on ice in the dark. Cells were washed 3 times with the CO2-independent medium, wells were scanned for IR800. From a separate plate with the “0 min.chase” wells the medium was aspirated and replaced with 20 µl of 2 × Laemmli sample buffer and lysed cells transferred to a PCR micro-titer plate (and stored on ice). For the other wells medium was replaced by the conditioned medium. Cells were transferred to 37 °C and 5% CO2 environment for 0 min, 90 min, 180 min or overnight. At these time points the cells were washed once, scanned for IR800 by Odyssey and subsequently lysed by 16 µl of 2 × Laemmli sample buffer and transferred to a PCR micro-titre plate. Collected samples were boiled for 4 min at 95 °C in a sealed PCR micro-titer plate to prevent evaporation. Lysate was separated on a SDS-PAGE gel of 15% for EGF, VHH and VLP_ADW_ and 10% for the gp140. Samples were Western blotted on a PVDF membrane. Actin was detected with mouse anti-actin (MP Biomedicals, 1/10,000) and donkey anti mouse peroxidase (DAMPO, 1/5000) followed by ECL, standard procedures.

### Chloroquine treatment

48-wells plates were coated with 0.25% gelatin and 6 × 10^4^ Her14 or 3T3 2.2 cells were seeded per well. Cells were incubated with indicated concentrations chloroquine for 60 min. 3T3 2.2 cells with 200 µM. Next, the cells were incubated with 13 nM EGF-IR800, for 15, 60 or 120 min. After washing cells were scanned with the Odyssey. Then medium was removed and cells were lysed with 2 × Laemmli sample buffer, boiled and analyzed on a 15% SDS page gel. IR800 was detected by Odyssey. After 60 min 100 µM chloroquine pretreatment, treatment with preincubated complexes (of gp140-IR800 with H2E1 and VLP_ADW_ with Hep1E1, respectively) was overnight.

### Assays HIV-1 binding to cells

For HIV-1 binding, cells were seeded as above. The HIV strains were pre-incubated with biheads as described above but with 30 ng virus + 60 ng biheads/200 µl conditioned medium. Where indicated heparin (Sigma) was added to 40 µg/ml for the last 30 min. of preincubation. Next the mixtures were added to the wells and plates were centrifuged for 10 min. 3000 rpm. and were incubated 3 h or 24 h while shaking (to mix properly). Cells were washed 4 × with PBS and cells lysed in 0.1% empigen in TBS and subjected to p24 Elisa. For the trans-infectivity assay after incubation for 3 or 24 h cells were washed 4 × with PBS and MT2 indicator cells (NIH Aids Reagent Program) were added 40,000 cells/200 µl/well in RPMI1640 + L-Glutamine (Lonza) supplemented with 10% fetal bovine serum (v/v)(Sigma), 10 µg/ml gentamycine (Life Technologies). 24 and 48 h later cytopathic effect (CPE) was scored. For pulse chase experiments cells were loaded with virus for 3 h and after washing conditioned medium was added for chasing for 24 h (or in some experiments in DMEM medium with 4%FBS, with similar result). 100 µM chloroquine (Sigma) treatment of cells started 2 h before adding virus and chloroquine kept was present during the 3 h loading. Chloroquine did not need to be re-added during 24 h chasing for inhibition of degradation (results not shown).

## Supplementary Information

Supplementary Information 1.

Supplementary Information 2.
