# Author Correction: Directing HIV-1 for degradation by non-target cells, using bi-specific single-chain llama antibodies

**DOI:** 10.1038/s41598-025-98744-z

**Published:** 2025-04-28

**Authors:** Jord C. Stam, Steven de Maat, Dorien de Jong, Mathia Arens, Fenna van Lint, Lavina Gharu, Mark H. van Roosmalen, Rob C. Roovers, Nika M. Strokappe, Ralf Wagner, Alexander Kliche, Hans J. de Haard, Paul M. van Bergen en Henegouwen, Monique Nijhuis, C. Theo Verrips

**Affiliations:** 1 Cell Biology, Neurobiology and Biophysics, Department of Biology, Science Faculty, Utrecht University, 3584 CH Utrecht, The Netherlands; 2 Translational Virology, Department of Medical Microbiology, University Medical Center Utrecht, Utrecht, The Netherlands; 3 Molecular Microbiology and Gene Therapy, Institute of Medical Microbiology and Hygiene, University of Regensburg, Regensburg, Germany; 4 Argenx, Industriepark Zwijnaarde 7, 9052 Zwijnaarde, Belgium; 5 QVQ Holding BV, Yalelaan 1, 3584 CL Utrecht, The Netherlands; 6 Present Address: Intervet, Wim de Körverstraat 35, 5831 AN Boxmeer, The Netherlands; 7 Present Address: LAVA Therapeutics, Yalelaan 60, 3584 CM Utrecht, The Netherlands

Correction to: *Scientific Reports* 10.1038/s41598-022-15993-y, published online 04 August 2022

In the original version of this Article, the ‘Competing interests’ section was incomplete.

“The authors declare no competing interests.”

now reads:

“The authors declare no competing interests. J.C. Stam has filed a patent application (GB202211100A·2022-07-29).”

The original Article has been corrected.

