# Supplementary figures and images for: Directing HIV-1 for degradation by non-target cells, using bi-specific single-chain llama antibodies
Source: Sci Rep. 2022 Aug 4;12:13413. doi: 10.1038/s41598-022-15993-y (PMC9352707; doi:10.1038/s41598-022-15993-y)

# Supplementary original Figures uncropped gels

Figure 2c, 2d, 3b and 3c

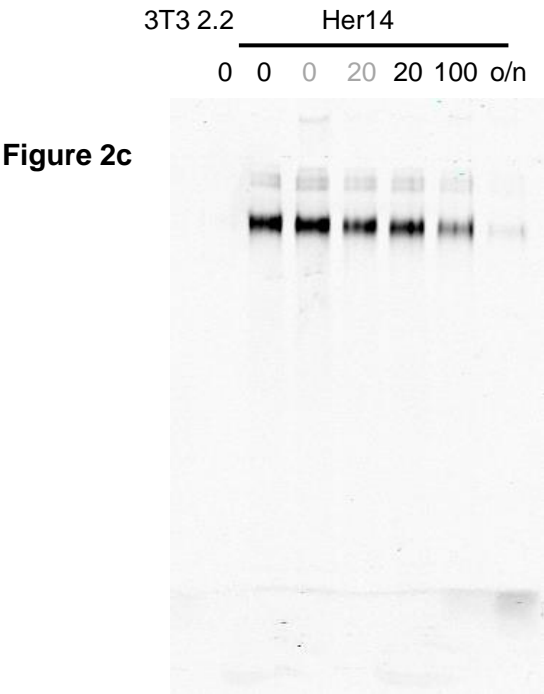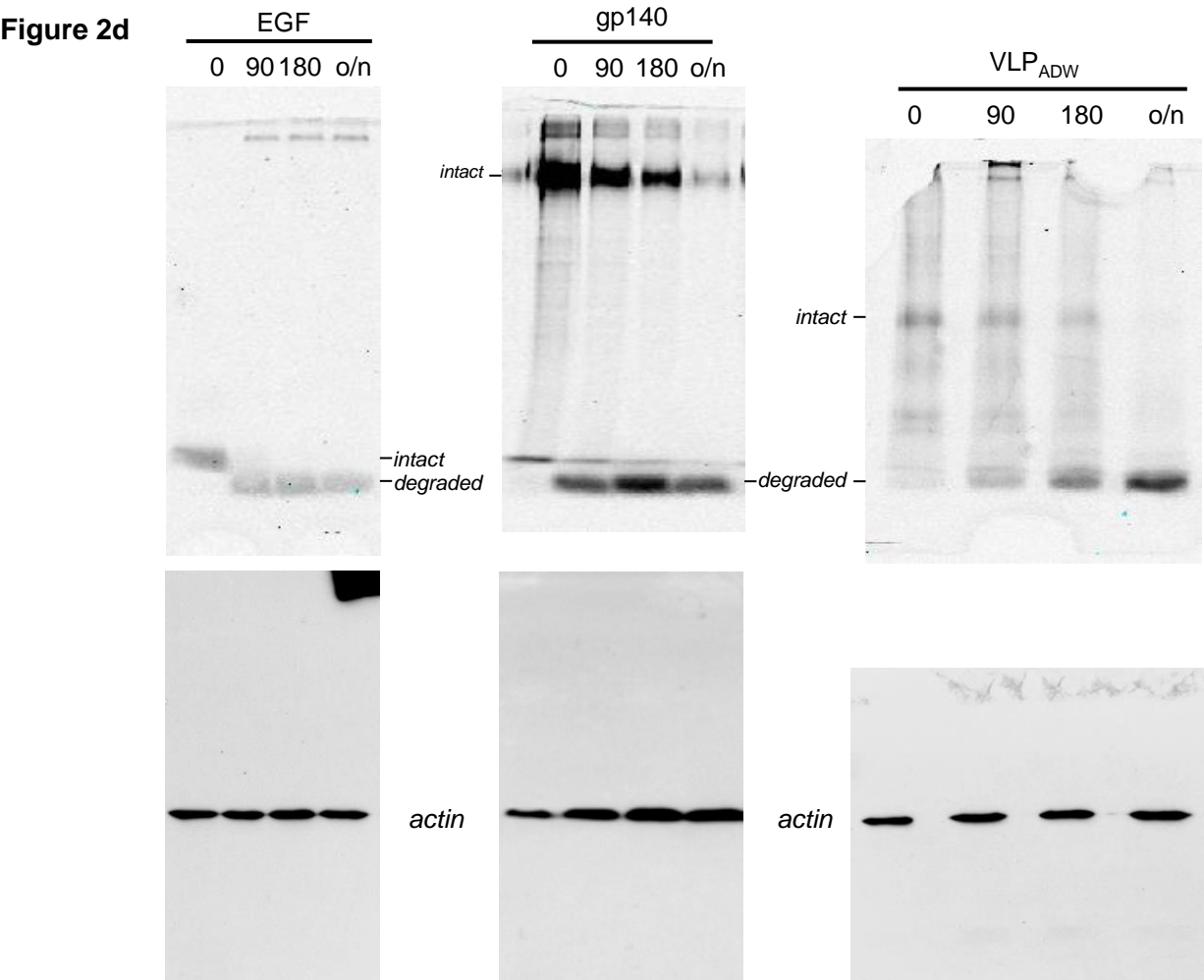

**Figure 3b**

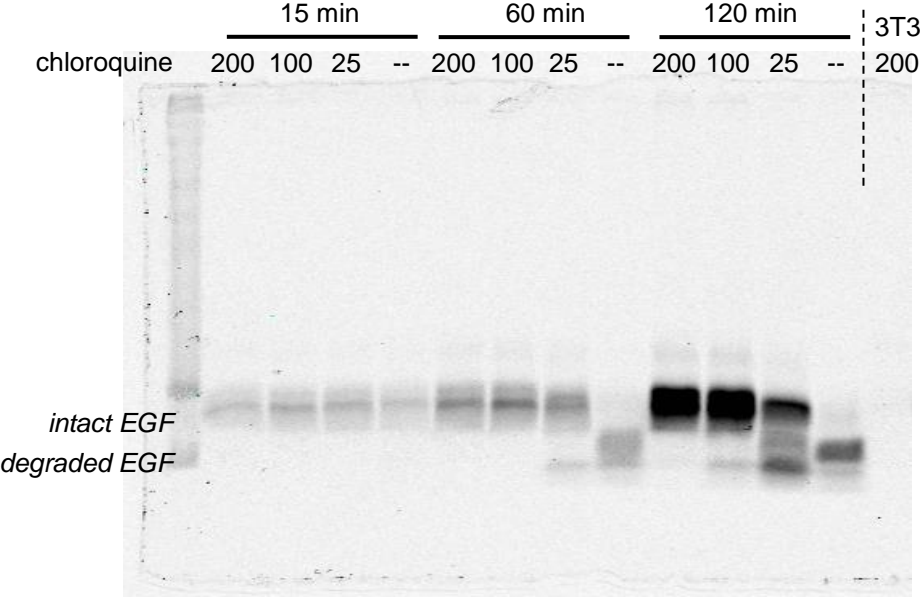

**Figure 3c**

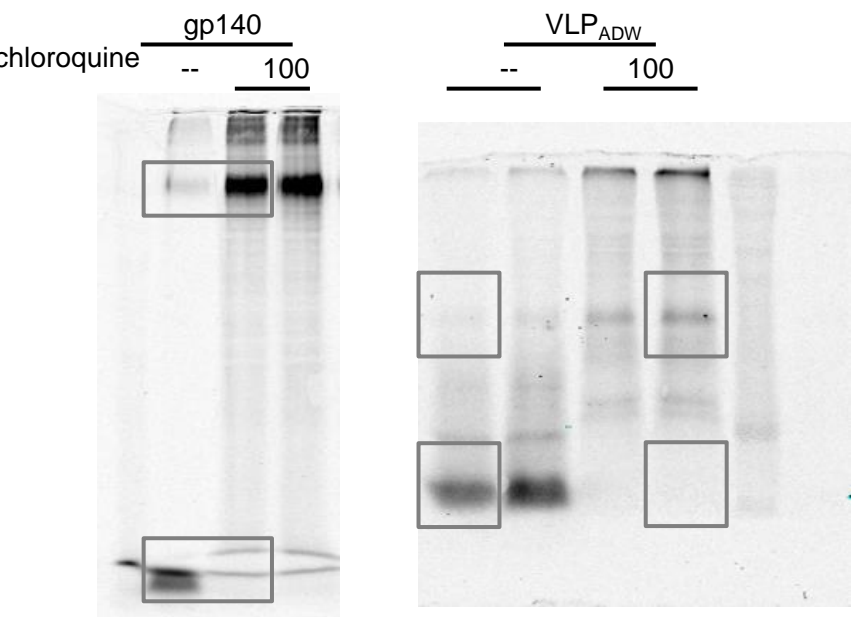

Supplement: Supplementary file 1 — Supplementary Information 1. [file 41598_2022_15993_MOESM1_ESM.pdf]
