# Supplementary material for: Directing HIV-1 for degradation by non-target cells, using bi-specific single-chain llama antibodies
Source: Sci Rep. 2022 Aug 4;12:13413. doi: 10.1038/s41598-022-15993-y (PMC9352707; doi:10.1038/s41598-022-15993-y)
Supplement: Supplementary file 2 — Supplementary Information 2. [file 41598_2022_15993_MOESM2_ESM.pdf]

Supplementary Figures

a.

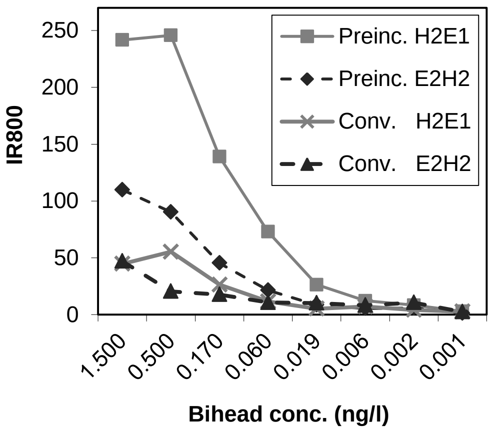

b.

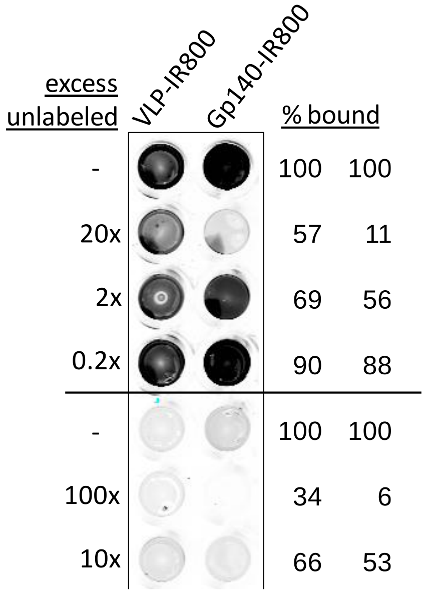

Supplementary Fig. S1. Avidity.

a. Preincubation of trimeric gp140-IR800 proteins with bispecific VHHs for 30 minute (Preinc) allows binding to three binding groups per trimer membrane protein and thus in a higher binding to coated EGFR than standard conventional stepwise FLISA (Conv). **b.** High avidity of VLPs that have multiple spikes. Antibodies added to viruses constitute preincubated complexes that will profit from avidity. To quantitatively investigate this, binding of preformed complexes was performed with and without excess competing antibodies. The excess antibodies will compete for EGFR binding, but VLP have higher avidity, due to synergistic binding of multiple biheads bound per virus. *Experiment.* EGFR was bound in wells, IR800-labeled VLP_{HBV} and gp140-UG were preincubated with corresponding bispecific VHHs (resp. Hep1E1 and H2E1) and the complexes were added together with the indicated amounts of unlabeled excess competing bispecific VHH containing the E1 moiety (resp. unlabeled H2E1 and unlabeled Hep1E1). In the upper panel 50 ng IR800-labeled proteins was used, in the lower panel 10 ng/well. *Result.* shows that the trimeric gp140-IR800 membrane proteins bind well in the presence of competing biheads, due to their higher avidity. This holds true even stronger for VLP with multiple attached biheads. Even with a 100 fold excess of competing biheads still 34% binding is observed (for the trimeric membrane proteins 6%). This is a favorable situation when viruses in the blood will be decorated with many antibodies.

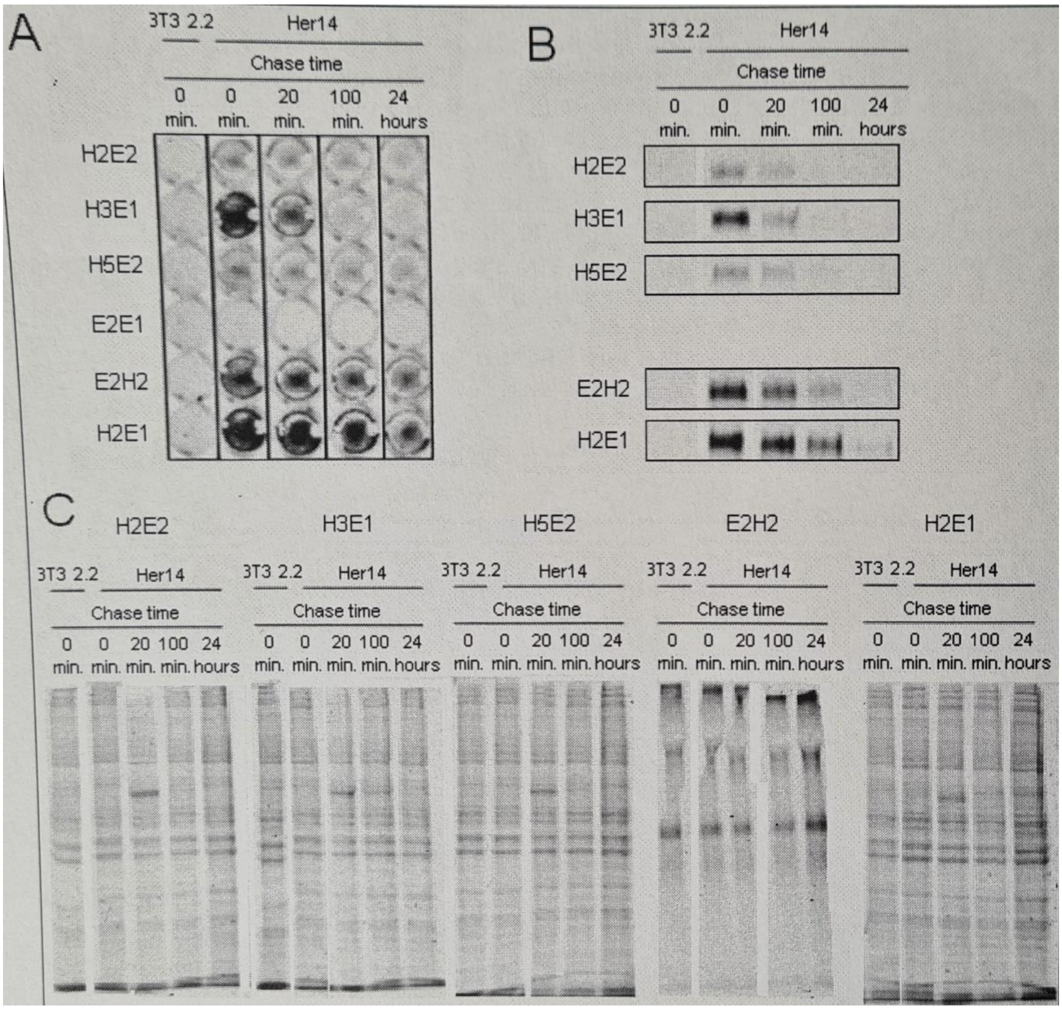

Supplementary Fig. S2. **Targeting HIV-1 proteins to EGFR-expressing Her14 cells results in binding and degradation.**

IR800-labeled gp140 (UG37) protein was preincubated with the indicated biheads and allowed to bind to Her14 cells and negative control 3T3 2.2 cells for 60 min on ice. Upon this pulse labeling a 0´ sample was taken and the other wells chased at 37°C for 20´, 100´ and 24 hrs. Samples were analysed in tissue culture wells (A) and on SDS-PAGE gels (B and C). ***A.*** IR800 signal, after washing, in wells at the indicated time-points. **B.** IR800 signal shows in protein gels the intact gp140-IR800 bound to the cells. **C.** demonstrates equal lysate protein loading on these same gels (CBB, Coomassie staining).

Conclusion: The bispecific VHHs H2E2, H3E1, H5E2, E2H2 and H2E1 all target IR800 labeld HIV-1 protein to the Her14 cells, resulting in rapid degradation. No signal was obtained with negative control cells 3T3 2.2 or control bihead E2E1.

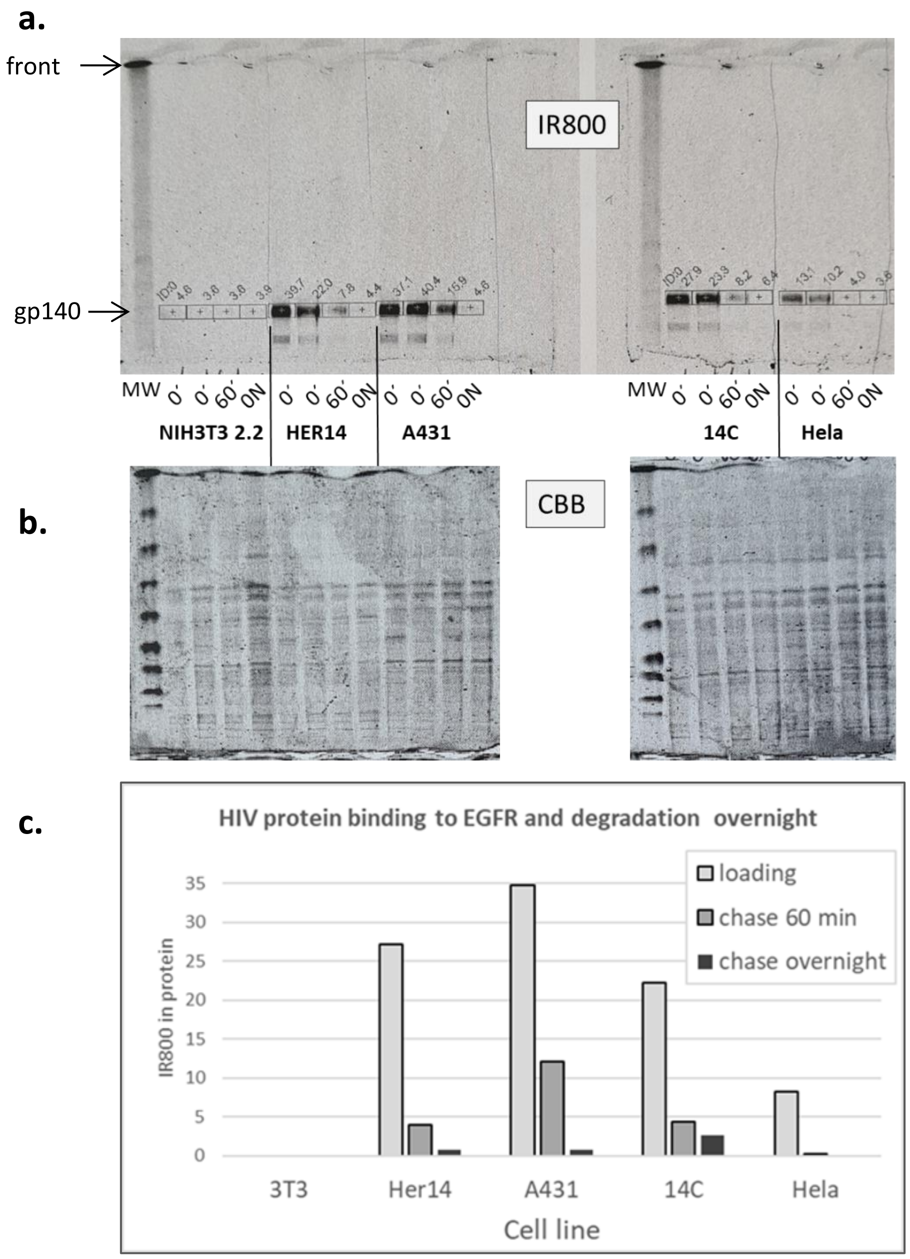

Supplementary Fig. S3. Targeting HIV-1 proteins to various EGFR-expressing cell lines results in binding and degradation.

IR800-labeled gp140 (UG37) protein was preincubated with bispecific VHH H3E1, and allowed to bind to the indicated cells for 60 min on ice. Upon this pulse labeling 2 samples were taken 0' and the other wells chased at 37 for 60 min and overnight. Samples were analysed on SDS-PAGE gels. *Similar results were obtained using bihead E2H2* ***a.*** the intact gp140-IR800 bound to the cells. **b.** shows equal lysate protein loading on the same gels (CBB, Coomassie staining). **c.** Graph quantifying bound intact gp140-IR800 during pulse-chase**.**

Experimental notes: the gels show low molecular weight on top *In the conditions applied here IR800 breakdown products have leaked out of the cells.*

Conclusion: Bound HIV-1 protein correlates to the (known) amount of EGFR on these cell types, NIH3T3 2.2 is negative control. During chase most of the bound gp140-IR800 is degraded.

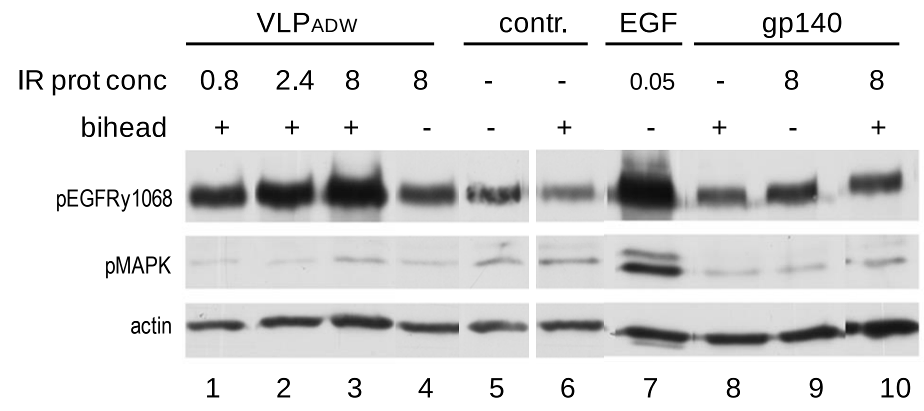

Supplementary Fig. S4. Bihead mediated targeting to EGFR does not activate signaling pathways implied in oncogenic signalling.

Monohead anti-EGFR E1 and E2 do not activate EGFR signaling, on the contrary, E1 inhibits EGF binding to the receptor[1]. Here activation of EGFR signaling was analysed upon bispecific VHH (bihead) mediated targeting. Phosphorylation of EGFR on Tyr1068, can be induced upon activation of the receptor and plays a role in internalization. More downstream phosphorylation of MAPK and Akt are involved in oncogenic signaling.

Experiment. IR800 labeled gp140 and VLP_{HBV} (concentration as indicated, in µg/ml) were preincubated resp. with 15 nM bihead H2E1 or Hep1E1 (+) or not (-). Incubation on cells was for 15 minutes. After washing cells were lysed and analysed by Westernblot with α-phospho EGFR y1068, α-phospho MAPK, and α-actin as primary antibodies. Westernblots were obtained from two gels as shown, within the gels some lanes have been removed.

Results. Whereas control EGF activates pEGFRy1068 and downstream pMAPK (lane 7), interestingly, bispecific VHH antibody mediated internalization of HIV proteins and VLPs differentially induce EGFR phosphorylation on tyrosine 1068. Binding of trimeric HIV proteins (gp140 + bihead) does not induce EGFR phosphorylation (lane 10), which is similar to the use of (multiple) conventional antibodies [2-5]. In contrast, targeted VLP_{HBV} dosis-dependently induced phosphorylation (lanes 1-3) and at 8 µg/ml almost as high as with EGF treatment, possibly due to plurivalent binding in VLPs. However, this did not lead to further downstream, potentially oncogenic, signalling: pMAPK was not activated above background and neither was Akt (data not shown). Possibly, VLPs with many binding sites might induce passive clustering of EGF Receptors (for a role of clustering see also ref.[6]), resulting in phosphorylation without concomitant downstream signalling towards MAPK. Whatever the mechanism, most importantly for our study we conclude that no oncogenic EGFR signaling was activated

References

1. Hofman, E. G. *et al*. EGF induces coalescence of different lipid rafts *J. Cell. Sci.* **121**, 2519 - 2528 (2008).

2. Friedman, L. M. *et al*. Synergistic down-regulation of receptor tyrosine kinases by combinations of mAbs: implications for cancer immunotherapy. *Proc. Natl. Acad. Sci. U. S. A.* **102**, 1915-1920 (2005).

3. Pedersen, M. W. *et al*. Sym004: a novel synergistic anti-epidermal growth factor receptor antibody mixture with superior anticancer efficacy *Cancer Res.* **70**, 588-597 (2010).

4. Oliveira, S. *et al*. Downregulation of EGFR by a novel multivalent nanobody-liposome platform. *J. Control. Release* **145**, 165-175 (2010).

5. Spangler, J. B. *et al*. Combination antibody treatment down-regulates epidermal growth factor receptor by inhibiting endosomal recycling. *Proc. Natl. Acad. Sci. U. S. A.* **107**, 13252-13257 (2010).
6. Heukers, R. *et al*. Endocytosis of EGFR requires its kinase activity and N-terminal transmembrane dimerization motif. *J. of Cell Science* **126**, 4900-4912 (2013)

Supplementary Methods

Supplementary Fig. S4 EGFR activation assays $1{,}6x10^5$ Her14 cells per well were seeded in gelatine coated 12 wells plate two days before the experiment and the next day serum starved with DMEM with 0.08% FBS, for 16 hours. The cells were incubated for 15 min with either nothing or 50 ng/ml EGF-IR800 or the indicated concentrations of IR800 labeled protein gp140, VLP_{HBV} or biheads Hep1E1, H2E1 or preincubated complexes of gp140-IR800 with H2E1 or VLP_{HBV}-IR800 with Hep1E1. After 15 min. incubation cells were washed with serum free medium and lysed with 35 µl of 4x Laemmli sample buffer. Samples were run on a 8% SDS-page gel and subsequently analysed by Western blot analysis with rabbit α-phopho-EGFR tyrosine 1068 (α-pEGFR), rabbit anti-phospho-p44/42 MAPK (Erk1/2) Thr202/Tyr204 (α-pMAPK) both from Cell Signaling Technology and mouse α-actin from MP Biomedicals as primary antibodies and DAMPO and GARPO as secondary antibodies. Detection was by standard enzyme chemiluminescence (ECL) methods.

Supplementary Table

a.

labeled reagent	EGF-IR800		VLP-IR800 (50 ng/well)			VLP-IR800 (150 ng/well)		
bihead	-	-	H2E1	-	-	H2E1	-	-
cells	H14	3T3	H14	3T3	H14	H14	3T3	H14
FCS	650	5	230	228	243	1020	1015	900
heparin	720	3	170	50	90	960	270	130

b.

	bispecific VHHs		control biheads		no bihead	**specific binding** Bispecific/control biheads
	H3E1	H5E2	E2E1	H5H4	no bihead	*(average of 2)*
no block	378	352	262	284	248	1,3 x
milk 4%	239	178	158	141	86	1,4 x
milk 2%	272	249	184	176	182	1,4 x
milk 1%	232	297	156	215	149	1,4 x
heparin 25	117	158	90	62	71	1,8 x
heparin 100	140	119	134	94	87	1,1 x

Supplementary Table S1. Attempts to block non-specific binding of HIV to cells.
a. Heparin blocking improves establishing bihead mediated binding of VLP_{HIV} to fibroblasts. To block nonspecific binding of HIV to cells, preincubated VLP_{HIV}–IR800 were bound to cells after different pretreatment conditions and IR800 measured. Result: FCS has no significant blocking effect. Heparin blocks nonspecific binding of HIV_{VLP} to cells without blocking EGF interaction with EGFR. With 150 ng/ml VLPs specific binding was higher than with 50 ng/ml VLPs, due to the lower molar concentration of VLPs. **b.** Bihead mediated targeting of infectious HIV, measured by p24 HIV ELISA. Result, milk does not block nonspecific interaction of infectious HIV with cells. Blocking with different concentrations of heparin improves the ratio specific binding but decreases total binding. In all cases bispecific VHH mediated binding is higher than non-specific binding with control VHHs.
